# Regulation of microRNA biosynthesis and expression in 2102Ep embryonal carcinoma stem cells is mirrored in ovarian serous adenocarcinoma patients

**DOI:** 10.1186/1757-2215-2-19

**Published:** 2009-12-16

**Authors:** Michael F Gallagher, Richard J Flavin, Salah A Elbaruni, Jamie K McInerney, Paul C Smyth, Yvonne M Salley, Sebastian F Vencken, Sharon A O'Toole, Alexandros Laios, Mathia YC Lee, Karen Denning, Jinghuan Li, Sinead T Aherne, Kai Q Lao, Cara M Martin, Orla M Sheils, John J O'Leary

**Affiliations:** 1 Department of Histopathology, University of Dublin, Trinity College, Institute of Molecular Medicine, St James's Hospital, Dublin 8, Ireland; 2 Department of Pathology, Coombe Women and Infants University Hospital, Dublin 8, Ireland; 3 Department of Obstetrics and Gynaecology, University of Dublin, Trinity College, Institute of Molecular Medicine, St James's Hospital, Dublin 8, Ireland; 4 The Centre for Molecular Oncologic Pathology, The Dana Faber Cancer Institute, Boston, MA02115, USA; 5 NUS Graduate School for Integrative Sciences and Engineering, National University of Singapore, Singapore 117456, Singapore; 6 Applied Biosystems, 850 Lincoln Centre Dr, Foster City, CA 94404, USA

## Abstract

**Background:**

Tumours with high proportions of differentiated cells are considered to be of a lower grade to those containing high proportions of undifferentiated cells. This property may be linked to the differentiation properties of stem cell-like populations within malignancies. We aim to identify molecular mechanism associated with the generation of tumours with differing grades from malignant stem cell populations with different differentiation potentials. In this study we assessed microRNA (miRNA) regulation in two populations of malignant Embryonal Carcinoma (EC) stem cell, which differentiate (NTera2) or remain undifferentiated (2102Ep) during tumourigenesis, and compared this to miRNA regulation in ovarian serous carcinoma (OSC) patient samples.

**Methods:**

miRNA expression was assessed in NTera2 and 2102Ep cells in the undifferentiated and differentiated states and compared to that of OSC samples using miRNA qPCR.

**Results:**

Our analysis reveals a substantial overlap between miRNA regulation in 2102Ep cells and OSC samples in terms of miRNA biosynthesis and expression of mature miRNAs, particularly those of the miR-17/92 family and clustering to chromosomes 14 and 19. In the undifferentiated state 2102Ep cells expressed mature miRNAs at up to 15,000 fold increased levels despite decreased expression of miRNA biosynthesis genes Drosha and Dicer. 2102Ep cells avoid differentiation, which we show is associated with consistent levels of expression of miRNA biosynthesis genes and mature miRNAs while expression of miRNAs clustering to chromosomes 14 and 19 is deemphasised. OSC patient samples displayed decreased expression of miRNA biosynthesis genes, decreased expression of mature miRNAs and prominent clustering to chromosome 14 but not 19. This indicates that miRNA biosynthesis and levels of miRNA expression, particularly from chromosome 14, are tightly regulated both in progenitor cells and in tumour samples.

**Conclusion:**

miRNA biosynthesis and expression of mature miRNAs, particularly the miR-17/92 family and those clustering to chromosomes 14 and 19, are highly regulated in both progenitor cells and tumour samples. Strikingly, 2102Ep cells are not simply malfunctioning but respond to differentiation specifically, a mechanism that is highly relevant to OSC samples. Our identification and future manipulation of these miRNAs may facilitate generation of lower grade malignancies from these high-grade cells.

## Background

Stem cell-like populations from multiple different malignancies can self-renew, differentiate and regenerate malignant tumours [1-9]. When introduced into SCID mice, a single so-called Cancer Stem Cell (CSC) is often sufficient to form a tumour representative of the original malignancy [8,10]. The phenotype of the resultant tumour can vary dramatically between malignancies but almost all CSCs generate tumours with populations of undifferentiated and differentiated cells. Tumours containing high concentrations of undifferentiated stem cells are considered to be highly malignant and differentiated tumours less malignant. We postulate that the differentiation capacity of the stem cell population within a malignancy may ultimately determine tumour grade. We aim to elucidate why stem cells have different differentiation potentials and generate tumours with different grades. Addressing this, we have chosen the embryonal carcinoma (EC) model, the only human stem cell model containing both pluripotent and nullipotent cells [11,12]. Pluripotent NTera2 EC cells differentiate into teratocarcinomas, three germ layer tumours containing a small proportion of undifferentiated stem cells [13]. In contrast, nullipotent 2102Ep EC cells can avoid differentiation during tumourigenesis, generating pure embryonal carcinomas, tumours consisting almost entirely of undifferentiated stem cells [14]. Thus this model allows comparative analysis of stem cell populations that generate highly and less malignant tumours through differing differentiation potentials. We postulate that the mechanisms facilitating tumourigenesis without differentiation may represent an avenue for targeting.

Ovarian cancer is the 8^th ^leading cause of cancer in women in the US and the leading cause of death from gynaecological malignancy in the western world [15]. Cancer of the ovary represents about 30% of all cancers of the female genital organs. About 205,000 cases of ovarian cancer are diagnosed worldwide each year [16]. Strikingly, stem cell-like populations linked to epithelial ovarian cancer (ovarian serous adenocarcinoma [OSC] is the most common histotype [17]; germ cell tumours of the ovary are rare) are anti-apoptotic and chemoresistant, suggesting a role in recurrent disease [18,19]. Significantly, EC is one of the most highly aggressive forms of ovarian malignancy, and intuitively, CSC-targeting is a potential avenue though which anti-cancer therapeutics can be advanced.

MicroRNAs (miRNAs) are short, non-coding RNAs that influence the transcription or translation of target mRNAs [20,21]. Primary miRNA transcripts (pri-miRs) are processed through stem-looped pre-miRs to achieve mature miRNAs, a process that is facilitated by the Drosha, Dicer and eIF6 proteins. Mature miRNAs hybridize at multiple locations along their target mRNA, including the seed region of the 3' untranslated region (UTR) [21]. In most cases, hybridization suspends these targets within the cell, preventing their translation. This post-transcriptional mechanism influences the timing at which mRNAs are presented for translation. At least one miRNA (miR-373) binds to a site in the promoter of its mRNA targets (E-Cadherin and CSDC2), acting as a positive regulator of transcription [22]. Specific miRNA populations have been described in stem cells, CSCs and malignancy in general [20,23,24]. Additionally, miRNAs located in specific clusters on chromosomes are often simultaneously synthesized. Simultaneous expression of miRNAs located in clusters along chromosomes 14 and 19 has been linked to ovarian malignancy [25,26] while the oncogenic role of the miR-17/92 family is well defined [27-29]. Aberrant expression of cancer-specific 'onco-miRs' is associated with the targeting of oncogenes and tumour suppressors in several different malignancies [30]. Many of these miRNAs have been shown to be vital to cancer cells [30].

In this study, we used realtime miRNA qPCR to individually, quantitatively analyze alterations in expression of all known miRNAs during early differentiation of NTera2 and 2102Ep EC cells. Using NTera2 cells as a model of a functionally differentiating EC cell, we aimed to identify miRNA mechanisms associated with the absence of differentiation associated with 2102Ep cells. Our data identifies key differences in expression levels of miRNA biosynthesis genes and of mature miRNAs, particularly those associated with the miR-17/92 family and clustering to chromosomes 14 and 19. Interestingly, undifferentiated 2102Ep cells express mature miRNAs at increased levels despite a decreased level of expression of Drosha and Dicer. While the malignancy-associated miR-17/92 cluster is prominent in both undifferentiated cell types, 2102Ep-specificity was associated with clustering to malignancy-associated chromosomes 19 and 14. Expanding this analysis we have identified NTera2- and 2102Ep-specific differentiation mechanisms that relate to miRNA biosynthesis and expression levels of mature miRNAs. Subsequently, we demonstrate that these mechanisms are similarly relevant to OSC patient samples. OSCs display up to 85% similarity with 2102Ep cells at the miRNA level. Our data reveals that miRNA regulation in 2102Ep EC cells is highly relevant to OSC samples.

## Methods

### Cell culture

Pluripotent NTera2 and nullipotent 2120Ep EC cells were a gift from Peter Andrews, University of Sheffield, and were maintained in the undifferentiated state in DMEM media supplemented with 10% FCS, 5% L-Glutamine and 5% PenStrep (Lonza, Basel, Switzerland). Differentiation was achieved by cell scraping (NTera2) or trypsinisation (2102Ep) and replating in the above cell culture media supplemented with 10 mM retinoic acid (RA) for 3 days.

### Case selection and tumour sample preparation

The training set comprised of 6 fresh frozen serous tumours (classified according to the FIGO system: stage (II-IV) and grade (2-3)) and normal whole ovary. Briefly, all tumour samples were taken from the ovary, snap frozen within 1 hour of surgery and stored at -80°C. After tissue processing in a cryostat at -20°C, frozen sections were cut, mounted on slides, stained with H&E and reviewed by a histopathologist (RJF) to confirm the original diagnoses and the presence of >70% tumour. For validation purposes, 40 ovarian serous carcinoma, classified as above, were selected from archival formalin-fixed, paraffin-embedded (FFPE) tissue, between the years 1991-2006 from St. James's Hospital, Dublin. H&E slides of all tumours were reviewed by a histopathologist (RJF) and original diagnoses confirmed. FFPE blocks were selected that contained over 90% tumour with contaminating stromal tissue estimated to be no more than 10%. 10 normal whole ovaries were used for normalisation.

### Isolation of RNA and TaqMan^® ^quantitative PCR (qPCR)

Total RNA was isolated from EC cells using the RNeasy kit (Qiagen, West Sussex, UK) and cDNA synthesised using the cDNA Archive Kit (Applied Biosystems {AB}, Foster City, CA), all per manufacturer's instructions. Differentiation status was confirmed through TaqMan^® ^qPCR analysis (AB) using pre-designed assays. Frozen tumour samples were placed in liquid nitrogen, ground thoroughly with a mortar and pestle and homogenized using a Qiashredder column (Qiagen). miRNA was isolated using the total RNA protocol, mirVANA™ microRNA isolation kit (Ambion, Austin, TX) as per manufacturer's instructions. Total RNA was extracted from FFPE material using RecoverAll™ Total Nucleic Acid Extraction Kit (Ambion Ltd., Cambridgeshire, UK) following the manufacturer's protocol. Quantitative miRNA realtime PCR analysis was carried out using the TaqMan^® ^microRNA assay early-access panel (AB) as per manufacturer's instructions. This panel included assays for each of the 330 human miRNAs known at the start of the study. The protocol detects mature miRNAs using looped-primer real time PCR involving three steps: reverse-transcription (RT), pre-PCR amplification and real-time PCR [31]. Each RT contained 10 ng total RNA. TaqMan^® ^analysis was carried out in triplicate on three biological replicate samples, using let-7a and miR-16 as both internal and negative controls, on the 7900 real time PCR system (AB).

### Data analysis, clustering and target gene prediction

miRNA data was generated using the ΔΔCT method [32]. Data was normalised through expression of let-7a. Hierarchal clustering was performed on the final miRNA expression lists using the Spotfire analysis^® ^platform (AB). Clustering was performed using the Unweighted Pair Group Method with Arithmetic Mean (UPGM). miRNA clustering analysis was carried out using the Sanger Institute's miRNA registry resource miRBase [33]http://microrna.sanger.ac.uk/sequences/ and the DIANA miRGen [34] miRNA resource. p-values with FDR correction were calculated using a t-test. All tests were two-tailed, and the significance level was set at P < 0.05.

## Results

### Undifferentiated 2102Ep EC cells display increased miRNA expression profiles, emphasizing high expression of chromosome 14 and 19 miRNAs

**Figure 1:**
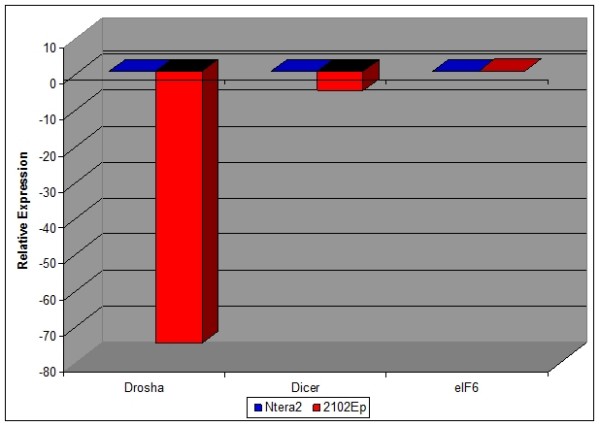
**Expression of miRNA biosynthesis genes and mature miRNAs in undifferentiated NTera2 and 2102Ep EC cells**. The relative expression levels of miRNA biosynthesis genes Drosha, Dicer and eIF6 in undifferentiated 2102Ep and NTera2 EC cells is shown. In each case, data represents expression in 2102Ep cells compared to NTera2. While the expression of eIF6 is almost identical, that of Dicer was downregulated slightly (4.3 fold) and of Drosha was substantially lower (74.5 fold) in undifferentiated 2102Ep EC cells compared to undifferentiated NTera2 EC cells.

The expression of miRNAs was assessed both globally, via the expression of miRNA biosynthesis genes Drosha, Dicer and eIF6, and individually, via quantitative miRNA qPCR analysis of a panel of 330 human miRNAs, which generates quantitative data for each mature miRNA as an individual assay. The relative expression of miRNA biosynthesis genes Dicer, Drosha and eIF6 in both undifferentiated EC cell types is shown in Figure 1. While eIF6 expression was almost identical, Dicer and Drosha were expressed at slightly and substantially lower levels in undifferentiated 2102Ep cells compared to undifferentiated NTera2 cells respectively. The same 203 miRNAs were expressed and 127 miRNAs undetected in both undifferentiated cell types, indicating strong (93.5%) qualitative similarity (Additional file 1). Undifferentiated 2102Ep cells uniquely expressed 21 additional miRNAs, representing a mechanism that is independent of NTera2 mechanisms. 10 (NTera2) and 9 (2102Ep) of the top 10 highest expressed miRNAs have previous associations with other malignancies (Table 1). 4 (NTera2) and 3 (2102Ep) of these miRNAs are members of the miR-17/92 family (miRs-17-5p, -19a, -19b, -106b and -25), a cluster associated with many malignancies. The 21 2102Ep-specific miRNAs prominently cluster to regions of chromosomes 19 and 14 (Additional file 1).

**Table 1:** The top 10 highest expressed miRNAs in undifferentiated NTera2 and 2102Ep cells, their expression levels and associations with other malignancies.

miRNA	Expression NTera2 (-dCt)	Malignancy	miRNA	Expression 2102Ep (-dCt)	Malignancy
		Prostate [50]			
**miR-222**	10.2	Leukaemia [51]	**miR-191**	12.7	Leukaemia [51]
**miR-19b**	9.7	Lymphoma [52]	**miR-302a**	11.8	
		Lymphoma [52]			Prostate [50]
**miR-19a**	9.6	Lung [53]	**miR-221**	11.1	Breast [59]
		Oesophageal [54]			Prostate [50]
**miR-103**	8.9	Pancreas [55]	**miR-222**	10.3	Leukaemia [51]
		Leukaemia [56]			Lymphoma [52]
**miR-17-5p**	8.6	Colorectal [57]	**miR-17-3p**	10.1	Colorectal [62]
		Prostate [58]			Oesophageal [54]
**miR-135b**	8.4	Breast [59]	**miR-103**	10.0	Pancreas [55]
**miR-25**	7.8	Gastric [60]	**miR-19b**	9.7	Lymphoma [52]
		Ovary [61]			Colorectal [57]
**miR-130a**	7.9		**miR-92**	8.8	Lymphoma [52]
		Colorectal [62]			Tumourigenesis
**miR-30c**	7.5	Ovary [61]	**miR-320**	8.6	[63]
		Gastric [60]			Prostate [50]
**miR-106b**	7.3	Prostate [58]	**miR-135b**	8.6	Breast [59]

**Figure 2:**
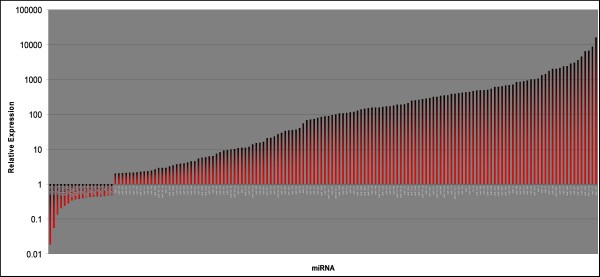
**The comparative expression levels of miRNAs in undifferentiated 2102Ep cells compared to undifferentiated NTera2 cells**. The relative expression of mature miRNAs in undifferentiated 2102Ep cells compared to Ntera2 cells is shown. Despite the qualitative similarities of the miRNA profiles expressed, our data indicated that the majority of miRNAs are expressed at higher levels in 2102Ep cells compared to NTera2.

**Figure 3:**
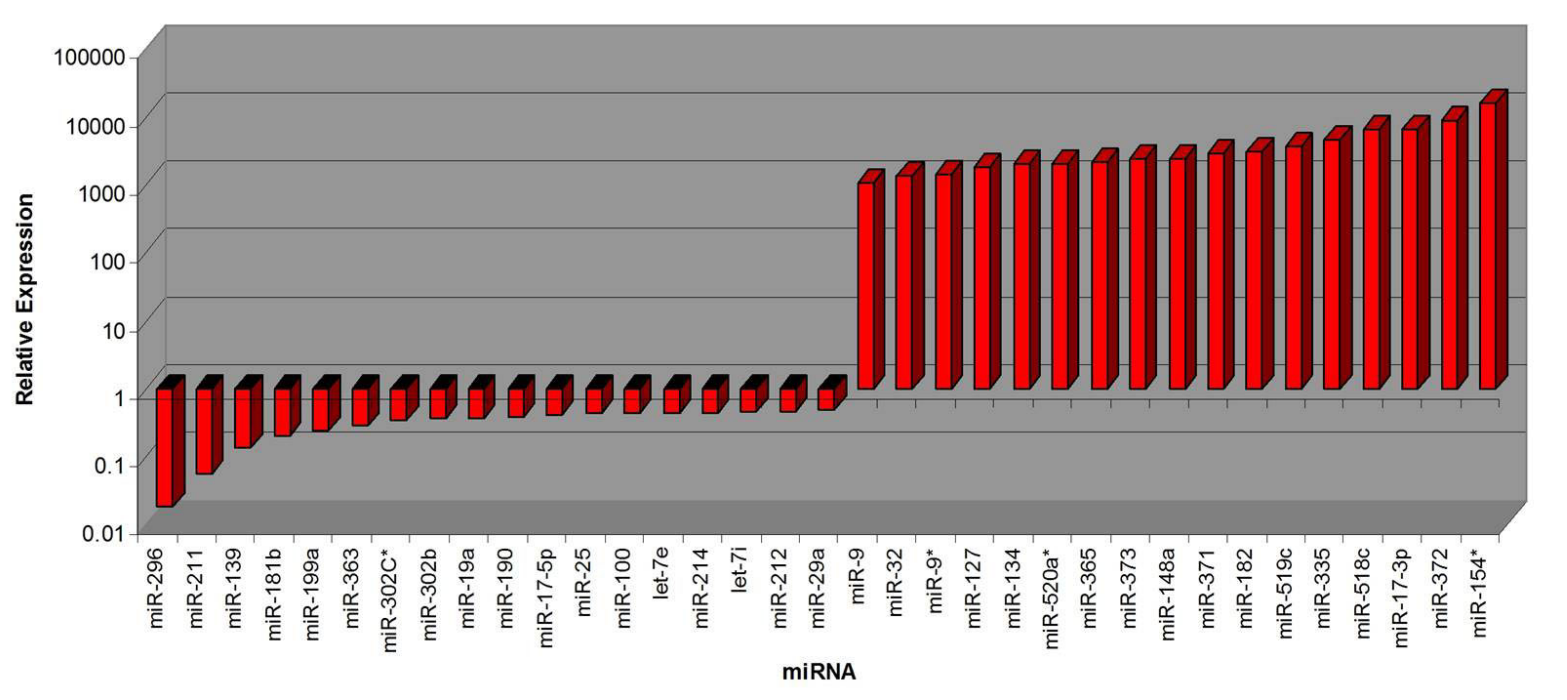
**The comparative expression levels of 17 of the highest upregulated and 18 of the highest downregulated miRNAs in undifferentiated 2102Ep cells compared to undifferentiated NTera2 cells**. The relative expression of the top mature miRNAs in undifferentiated 2102Ep cells compared to Ntera2 cells is shown These results demonstrate a substantial bias towards increased expression of mature miRNAs in 2102Ep cells. The miRNAs and levels of expression are listed in Additional file 1.

Despite these qualitative similarities, a quantitative comparison identified substantial differences. Our analysis reveals that the qualitative similarities of undifferentiated NTera2 and 2102Ep cells are associated with the miR-17/92 family. In contrast substantial quantitative differences between the cells are associated with clustering to chromosomes 14 and 19. 134 of the 203 miRNAs were expressed at higher levels in 2102Ep cells compared to NTera2 cells while 18 were downregulated (Figure 2, Additional file 1). 17 miRNAs were particularly notable, displaying 1,000-15,000 fold higher expression in 2102Ep cells, while 18 miRNAs showed decreased expression of up to -53 fold (Figure 3). The majority of these 17 upregulated and 18 downregulated miRNAs have previous associations with malignancy (Additional file 2). Prominent clustering to chromosomes 14 and 19 was apparent (Additional file 1). Additionally, 7 of these miRNAs are members of the miR-17/92 cluster (miRs-17-5p, -17-3p, 18a*, -18b, -92, -106a and-363) and were up to 6,000 fold higher expressed in undifferentiated 2102Ep cells (Tables 1, 2).

### Regulation of miRNA expression by differentiated NTera2 cells is absent in 2102Ep cells

**Figure 4:**
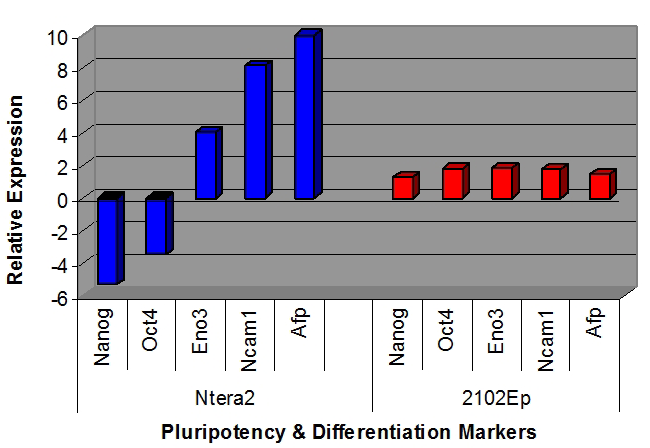
**Differentiation status and miRNA biosynthesis gene expression in differentiated NTera2 and 2102Ep cells**. The expression of markers of pluripotency (Oct4 and Nanog) and of endoderm (Afp), ectoderm (Ncam1) and mesoderm (Eno3) differentiation is shown. In each case, data represents changes in expression in the differentiate state compared to undifferentiated state. In NTera2 cells, differentiation status is confirmed by decreases in expression of Oct4 and Nanog and increases in expression of Afp, Ncam1 and Eno3. 2102Ep cells alter expression of these genes less than two-fold, confirming nullipotency.

**Figure 5:**
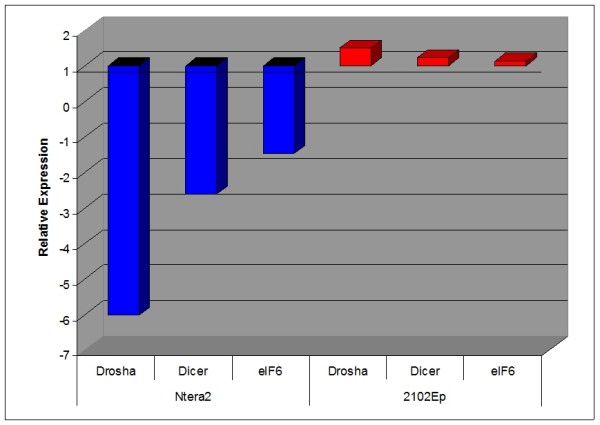
**The expression levels of the miRNA biosynthesis genes Drosha, Dicer and eIF6 in differentiated cells compared to undifferentiated cells**. Expression of Drosha decreases 65 fold while that of Dicer decreases 2.6 fold and of eIF6 decreased less than 2 fold upon differentiation of NTera2 cells. In contrast, the level of differential expression of each gene is within 1.0 fold in both undifferentiated and differentiated 2102Ep cells. Maintained expression of miRNA biosynthesis genes, therefore, is associated with the 2102Ep nullipotent phenotype.

We next treated both cell types with retinoic acid (RA) for 3 days to induce differentiation. Data is presented as the alteration of expression in differentiated cells compared to undifferentiated cells. This time point was chosen to assess miRNA expression in early differentiation. Differentiation status of RA-treated NTera2 cells was confirmed by decreased expression of pluripotency markers Oct4 and Nanog and increased expression of differentiation markers Ncam1, Eno3 and Afp (Figure 4). While eIF6 expression was unaltered, that of Drosha and Dicer was slightly decreased in differentiated NTera2 cells (Figure 5). 113 miRNAs displayed altered expression in differentiated NTera2 cells compared to undifferentiated cells (Figure 3, Additional file 3). Of these, 65 miRNAs were upregulated and 48 downregulated (Additional file 3). The majority of the top 10 upregulated and downregulated miRNAs in differentiated NTera2 cells have previous associations with other malignancies (Table 2). In contrast to undifferentiated cells, there is no overlap between top tens in each cell type and no prominence of miR-17/92 miRNAs is present (Additional file 3).

**Table 2:** The top 10 highest expressed miRNAs in differentiated NTera2 and 2102Ep cells, their expression levels and associations with other malignancies.

miRNA	Relative Expression	Malignancy	miRNA	Relative Expression	Malignancy
**Downregulated NTera2**			**Downregulated 2102Ep**		
					Tumourigenesis [63]
**miR-507**	-100,000		miR-518c*	-634.0	
**miR-142-5p**	-100,000	Lung [64]	**miR-153**	-30.9	
					Lung [77]
**miR-520b**	-100,000		**let-7g**	-10.1	Colon [78]
**miR-522**	-100,000		**miR-504**	-9.9	
**miR-122a**	-100,000		**miR-362**	-8.1	
					Lymphoma [52]
**miR-515-5p**	-100,000		**miR-17-3p**	-6.2	Colorectal [62]
miR-182*	-100,000	Prostate [65]	**miR-511**	-3.4	Liver [47]
miR-199a*	-100,000	Lung [66]	**miR-193b**	-3.2	Endometrial [72]
		Mesothelioma [67]			
**miR-7**	-100,000	Lung & Breast [68]	**miR-455**	-2.8	
**miR-206**	-100,000	Breast [69]	**miR-431**	-2.7	
**Upregulated NTera2**			**Upregulated 2102Ep**		
		Kaposi Sarcoma [70]			Medullablastoma [79]
**miR-140**	6.1	Breast [30]	**miR-199b**	2.6	
		Breast [59]			Lymphoma [80]
miR-191*	6.7	Leukaemia [51]	**miR-363**	2.6	
**miR-188**	8.3		**miR-129**	3.0	Gastric [81]
		Endometrial [71]			Tongue [78]
**miR-99b**	11.1		**miR-184**	3.8	Neural [82]
**miR-509**	18.3		**miR-519d**	3.8	
		Tongue [72]			Breast [75]
**miR-219**	21.9		**miR-10a**	119.2	Leukaemia [83]
		Lung [73]			
**miR-99a**	22.5	Ovary [35]	**miR-433**	100000	
		Ovary [61]			Glioblastoma [84]
**miR-335**	26.0	Myeloma [74]	**miR-425**	100000	
		Breast [75]			Multiple [85]
**miR-10a**	100000	Leukaemia [51]	**miR-105**	100000	
		Prostate [76]			Glioblastoma [86]
**let-7c**	100000	Lung [52]	**miR-137**	100000	Melanoma [87]

**Figure 6:**
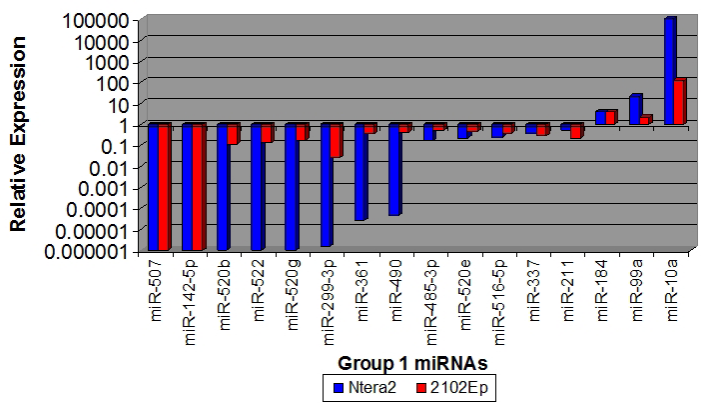
**Comparison of the expression levels of miRNAs altered in differentiated NTera2 and 2102Ep cells: Group 1 miRNAs**. miRNAs were grouped according to their expression patterns upon differentiation of NTera2 and 2102Ep cells. 15 Group 1 miRNAs are similarly altered in both cell types. We propose that Group 1 miRNAs likely act upstream of any lesion in the 2102Ep differentiation mechanism. miRNAs in each group are listed in Additional file 3.

**Figure 7:**
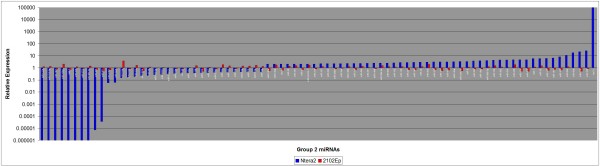
**Comparison of the expression levels of miRNAs altered in differentiated NTera2 and 2102Ep cells: Group 2 miRNAs**. miRNAs were grouped according to their expression patterns upon differentiation of NTera2 and 2102Ep cells. 85 Group 2 miRNAs are altered in NTera2 cells but unaltered in 2102Ep cells. We propose that Group 2 miRNAs likely act downstream of any lesion in the 2102Ep differentiation mechanism. miRNAs in each group are listed in Additional file 3.

**Figure 8:**
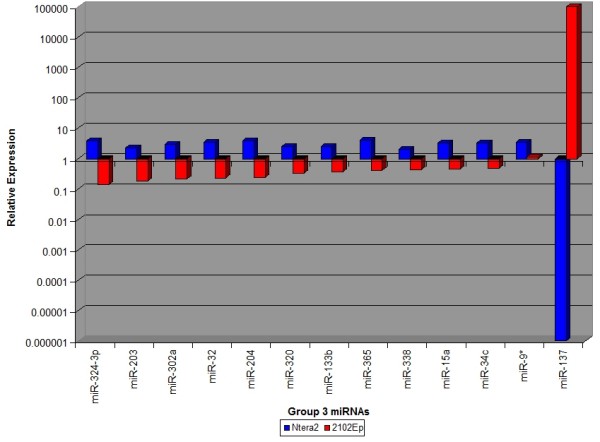
**Comparison of the expression levels of miRNAs altered in differentiated NTera2 and 2102Ep cells: Group 3 miRNAs**. miRNAs were grouped according to their expression patterns upon differentiation of NTera2 and 2102Ep cells. 13 Groups 3 miRNAs are altered in an opposite fashion in each cell line. Group 3 miRNAs represent a 2102Ep-specific response to differentiation that is independent of NTera2 mechanisms. miRNAs in each group are listed in Additional file 3.

**Figure 9:**
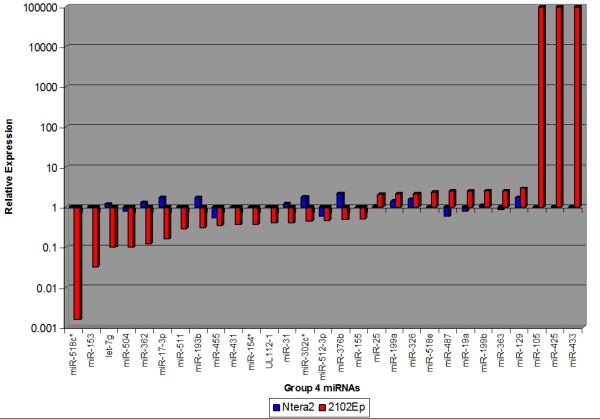
**Comparison of the expression levels of miRNAs altered in differentiated NTera2 and 2102Ep cells: Group 4 miRNAs.** 29 Group 4 miRNAs are altered in RA-treated 2102Ep cells but unaltered in differentiated Ntera2 cells. Group 4 miRNAs represent a 2102Ep-specific response to differentiation that is independent of Ntera2 mechanisms. miRNAs in each group are listed in Additional file 3.

We next assessed the regulation of these 113 miRNAs in 2102Ep cells treated with RA. We reasoned that the response of 2102Ep cells to RA could reveal mechanisms associated with this cell line's ability to remain undifferentiated during tumourigenesis. Unaltered expression of pluripotency and differentiation markers confirmed nullipotency of 2102Ep cells (Figure 4). The results demonstrate that high grade 2102Ep cells are associated with unaltered expression of most miRNAs that are altered during NTera2 differentiation. In contrast to NTera2 cells, levels of eIF6, Drosha and Dicer expression were not altered in differentiated 2102Ep cells (Figure 5). Based on their expression in 2102Ep cells, we have placed these 113 miRNAs into 4 Groups (Figures 6, 7, 8 and 9 & Additional file 3). Group 1 miRNAs are expressed similarly in each cell type. Group 2 miRNAs are altered by differentiation treatment in NTera2 cells but are unaltered in 2102Ep cells. Groups 3 and 4 miRNAs are described in the next section. There are 16 miRNAs in Group 1 and 84 miRNAs in Group 2. 3 and 4 Group 1 miRNAs cluster to chromosomes 14 and 19 respectively (Figure 6 & Additional file 3). 7 Group 2 miRNAs cluster to chromosome 14 and 16 to chromosome 19 (Figure 7 & Additional file 3). Thus, Group 1 miRNAs represent a common mechanism while Group2 miRNAs are NTera2-specific.

### 2102Ep cells respond independently to retinoic acid treatment

During our analysis we identified a third and fourth group of miRNAs that represent a 2102Ep-specific response to differentiation (Figures 8 and 9 & Additional file 3). Group 3 miRNAs are altered in both differentiated cell types but in an opposite fashion. Group 4 miRNAs are altered in 2102Ep cells following RA treatment but not in NTera2 cells. These groups constitute a specific 2102Ep response to differentiation that is independent of NTera2 mechanisms. 12 Group 3 miRNAs are downregulated while only one, miR-137, is upregulated in 2102Ep cells. No Group 3 miRNAs cluster to regions of chromosomes 14 and 19. Group 4 contains 29 miRNAs. 17 Group 4 miRNAs are downregulated and 12 upregulated. Downregulated miRNAs range in expression to decreases of -633 fold. 3 miRNAs, miRs-433, -425 and -105, are only expressed in differentiated 2102Ep cells. 5 Group 4 miRNAs cluster to chromosome 14 and 3 to chromosome 19 (Additional file 3). Once again, the majority of Group 3 and 4 miRNAs have previous associations with malignancy (Additional file 4). While Group 2 miRNAs represent an absence of regulation in differentiated 2102Ep cells, Groups 3 and 4 represent specific responses by differentiated 2102Ep cells that are independent to the response of differentiated NTera2 cells. Finally, the previously discussed group of 21 miRNAs that were expressed in undifferentiated 2102Ep cells but not in NTera2 cells remain unaltered upon RA treatment of 2102Ep cells. These 21 miRNAs represent an independent miRNA mechanism employed by 2102Ep cells in both states. Their prominent clustering to regions of chromosomes 14 and 19, which are associated with ovarian cancer, is striking (Additional file 1).

### miRNA expression in high-grade OSC samples

**Figure 10:**
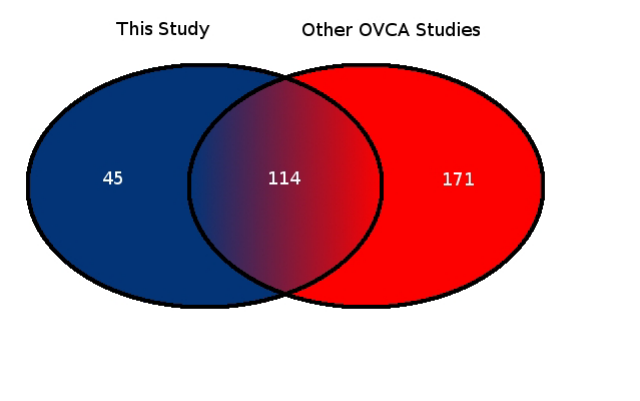
**Comparison of miRNA expression in OSC patient samples**. The venn diagram shows the number of differentially expressed miRNAs identified in the current study and the number of miRNAs identified in six previous studies [26,36-40]. These overlap substantially.

**Figure 11:**
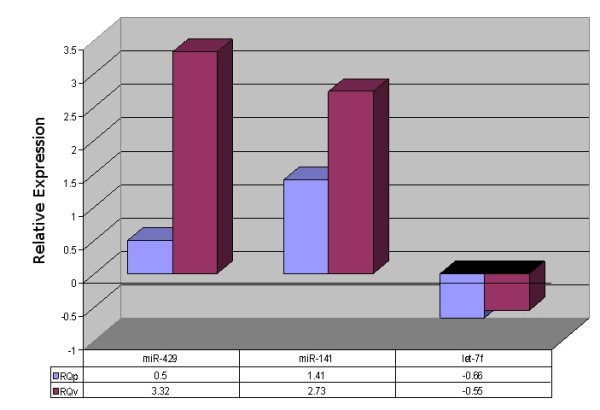
**Validation of OSC patient sample miRNA data**. Validation of training set: Log10 RQ values for miR-429, mir-141 and let-7f in both the pilot study (RQp) and the larger independent FFPE cohort (RQv). There was modest correlation between both sets of results (r = 0.50).

**Figure 12:**
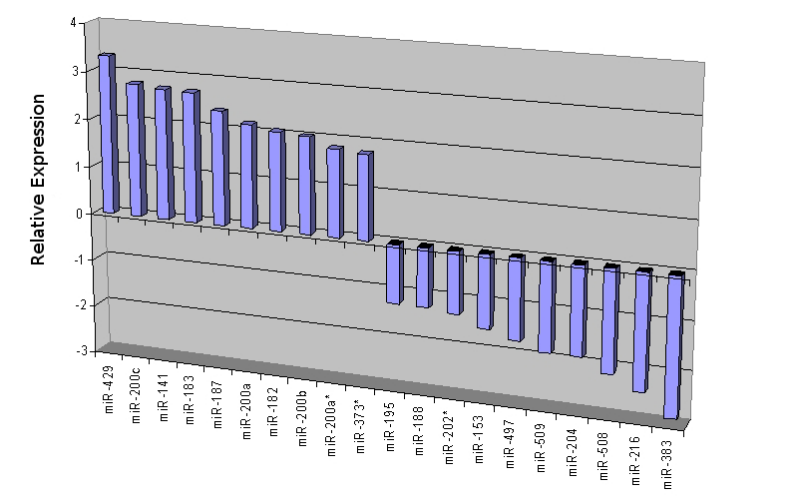
**Top 10 miRNAs up- and downregulated in OSC patient samples**. Barchart of the mean logarithmic fold change (RQ) of the top ten up and downregulated miRNAs in OSC samples relative to normal ovary.

We have previously reported increased expression of Dicer and eIF6 in high-grade OSC samples compared to normal [35]. Here, the expression of 330 miRNAs in high-grade OSC samples was assessed as above. 154 miRNAs (35 up- and 119 downregulated) were specifically expressed in OSCs compared to matched non-malignant ('normal') ovarian tissue samples (Additional file 5). Our tumour sample data shows 72% concordance with previously published ovarian tumour data (Figure 10[26,36-40]). A subset of miRNAs was further validated in a larger independent cohort of OSC samples (Figure 11). This indicated that our data is a good representative data set. The top 10 up- and downregulated tumour-specific miRNAs are illustrated in Figure 12. All but 2 of which have previous associations with malignancy (Table 3). 4 members of the miR-17/92 family are downregulated (miRs-17-3p, -18a*, -20a and -92) but only 1 upregulated (miR-18a) in OSC samples. Additionally, the prominence for clustering to chromosome 14 was maintained in OSC samples while that to chromosome 19 was decreased (Additional file 5). We initially compared our OSC miRNA data to that of undifferentiated EC cells. 106 of the 203 (52%) miRNAs commonly expressed by both undifferentiated EC cell types were OSC-specific (Additional file 5). Of this 52%, 86 miRNAs were upregulated and 19 downregulated, indicating a bias towards upregulation of EC-specific miRNAs in OSC samples. miR-17/92 family members expressed in OSC samples are similarly expressed in EC cells. 10 of the 21 (48%) 2102Ep-specific miRNAs were similarly OSC-specific (Additional file 5). The prominence for clustering to chromosome 14 was maintained in OSC samples while that to chromosome 19 is lost.

**Table 3:** The top 10 highest expressed miRNAs in OSC patient samples compared to normal ovary, their expression levels and associations with other malignancies.

miRNA	Relative Expression	Malignancy
**Downregulated**		
**miR-383**	-1000	
**miR-216**	-250	Pancreas [88,89]
**miR-508**	-142.9	
		Leukaemia [55]
**miR-204**	-71.5	Pancreas [88]
**miR-509**	-67.1	
		Gastric [89]
**miR-497**	-47.6	Breast [90]
**miR-153**	-33.3	
miR-202*	-19.6	Leukaemia [91]
**miR-188**	-17.2	
		Liver [92]
**miR-195**	-16.9	Bladder [93]
**Upregulated**		
**miR-429**	2082	Ovary [37,94]
		Metastasis [95]
**miR-200c**	600	Pancreas [96]
		Metastasis [95]
**miR-141**	533	Gastric [97]
		Lung [98]
**miR-183**	510	Colorectal [54]
**miR-187**	237	Thyroid [99]
**miR-200a**	145	Ovary [36,94]
**miR-182**	113	Prostate [83]
		Metastasis [95]
**miR-200b**	103	Pancreas [96]
miR-200a*	67	Ovary [38,94]
		Oesophageal [100]
**miR-373**	59	Leukaemia [51]

### Relevance of OSC-specific miRNAs to EC cells

**Figure 13:**
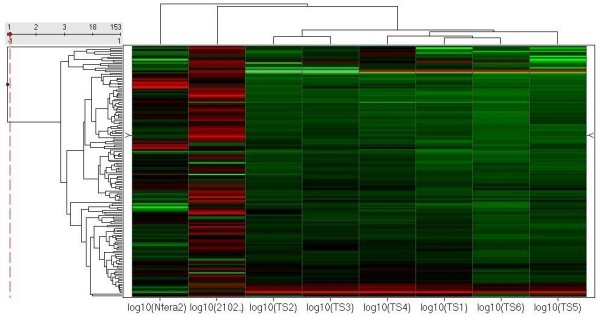
**Comparison of miRNA expression in EC cells and OSC patient samples**. Hierarchal clustering of the differentially expressed miRNAs in EC cells and ovarian tumour samples is shown. Hierarchal clustering was performed on miRNAs altered upon differentiation of nullipotent (2102Ep) and pluripotent (NTera2) EC cells and in ovarian tumour samples (TS) compared to normal tissue. Clustering indicates that alterations in miRNA expression during differentiation of the nullipotent EC cell type are more similar to tumour samples than alterations in miRNA expression during differentiation of the pluripotent cells.

OSC miRNA data was next compared to differentiated EC miRNA data. Hierarchal clustering indicated that OSC samples clustered with 2102Ep cells while NTera2 cells were more divergent (Figure 13). This is in concordance with the highly aggressive phenotype of 2102Ep EC cells but may also be related to lineage-specificity. Approximately 85% of OSC-specific miRNAs were expressed in 2102Ep cells (Additional file 5). The majority of tumour-specific miRNAs (whether up- or downregulated in tumours) were consistently expressed in 2102Ep cells. The next largest overlap was observed for tumour-specific miRNAs that were downregulated in 2102Ep cells. Comparatively few tumour-specific miRNAs were upregulated in 2102Ep cells. This indicates that tumour-specific miRNAs are tightly regulated in 2102Ep cells. We next asked whether the top 10 up- and downregulated OSC-specific miRNAs play a role in 2102Ep cells. All but 2 (miRs-202* and -216) top downregulated tumour-specific miRNAs were detected in 2102Ep cells. All of the top upregulated tumour-specific miRNAs were detected in 2102Ep cells.

We subsequently assessed the relevance of Group 1 2, 3 and 4 miRNAs to OSC samples. Approximately half of Group 1 and 2 miRNAs were found to be OSC-specific, the majority of which were downregulated in OSC samples (Additional file 3). Prominent clustering to chromosome 14 is maintained in OSC samples but is substantially decreased for chromosome 19. 62% of Group 3 miRNAs and 38% of Group 4 miRNAs were OSC-specific, again showing a bias towards downregulation of miRNAs (Additional file 3). Clustering to chromosomes 14 and 19 was decreased for Group 4 miRNAs expressed in OSC samples. This identifies a substantial group of miRNAs that are regulated in both EC cells and OSC samples. Regulation of Group 3 miRNAs was particularly relevant to OSC samples.

## Discussion

Compromising the ability of CSCs to remain in the undifferentiated state is a potential avenue for anti-cancer therapies. Before this can be achieved, we must identify mechanisms involved. 2102Ep cells are the stem cell population of ECs, malignant tumours that can arise in the ovary [11]. *In vivo*, these cells avoid differentiation to produce highly-aggressive, poorly-differentiated tumours [11]. In this study we report that miRNA regulation is associated with this phenotype. In undifferentiated cells, this involves decreased expression of miRNA biosynthesis genes, increased expression of mature miRNAs and expression of miRNAs clustering along chromosomes 14 and 19. When treated with RA, 2102Ep cells avoid differentiation and continue to proliferate. This is associated with consistent levels of expression of miRNA biosynthesis genes and mature miRNAs while expression of miRNAs clustering to chromosomes 14 and 19 is deemphasised. OSC samples displayed decreased expression of miRNA biosynthesis genes, decreased expression of mature miRNAs and prominent clustering to chromosome 14 but not 19. This indicates that miRNA biosynthesis and levels of miRNA expression, particularly from chromosome 14, are tightly regulated both in progenitor cells and in tumour samples.

Mechanistically, our data indicates that undifferentiated 2102Ep EC cells can express more miRNAs at higher levels of expression than NTera2 cells despite their decreased expression of miRNA biosynthesis genes. We have previously described the association of decreased eIF6 expression with high-grade OSC samples from patients with reduced disease-free survival [35]. In concordance with this, decreased Dicer and Drosha expression is linked to advanced stage ovarian cancer and increased expression to increased patient survival [41]. Interestingly, eIF6 expression was unaltered in EC cells. This indicates the complexity of miRNA biosynthesis regulatory mechanisms in EC stem cells and tumours. This mechanism is clearly linked to higher grade malignancy and its elucidation will be the subject of ongoing analysis. The levels of expression of miRNAs were higher in undifferentiated 2102Ep cells than NTera2 cells. 2102Ep cells express 21 miRNAs in both states that are not expressed by NTera2 cells. Similarly, OSC samples showed biased upregulation of miRNAs compared to non-malignant samples. Thus levels of mature miRNA expression are tightly controlled both in progenitor cells and developed tumours. It is widely reported that specifically regulated miRNA groups commonly occur in clusters on specific chromosomes. Prominent clustering to three particular sites was observed in this study: the miR-17/92 cluster and chromosomes 14 and 19, which have been linked with numerous malignancies. miR-17/92 family clusters are associated with regulation of proliferation, angiogenesis and apoptosis in malignancy [27-29]. These miRNAs were highly expressed by both undifferentiated cell types and were not prominently 2102Ep specific. Previous associations of chromosome 19 with germ cell tumours and of chromosome 14 with ovarian cancer are particularly striking [26,42]. miRNAs with 2102Ep specificity prominently clustered to these chromosomes while Group 1 miRNAs did not. miRNAs in these regions may contribute to the 2102Ep phenotype and will be assessed by ongoing analysis.

2102Ep cells avoid differentiation through a mechanism that involves maintained expression of pluripotency master genes Oct4 and Nanog [11]. We have identified miRNA regulation mechanisms associated with this phenotype. Group 1 miRNAs behave similarly in each EC cell type and are thus likely to act upsteam of the 2102Ep differentiation lesion. Group 2 miRNAs are altered upon differentiation of NTera2 cells but not in 2102Ep cells, suggesting that their role lies downstream of the 2102Ep differentiation lesion. It is possible that Group 1 miRNAs are involved with initiation of tumourigenesis from EC cells. For example, miR-10a targets HoxA1, a long established marker of undifferentiated EC cells [43]. Approximately half of these miRNAs were OSC-specific, indicating that both groups are relevant to tumour biology. This may be reflective of the heterogeneous nature of tumour samples, which contain a spectrum of differentiating cell types. Our data indicates that unaltered expression of Group 2 miRNAs is associated with the ability of 2102Ep cells to remain in the undifferentiated state in the presence of a differentiation signal. Maintenance of these miRNAs may protect these EC cells from differentiation signals *in vivo*. This is supported by their reported validated targets. For example, differentiation regulators are targeted by miRs-199a (Bmp2) and -206 (MyoD) [44,45]. The future characterisation and manipulation of this lesion may facilitate generation of lower grade tumours from 2102Ep cells.

The substantial overlap between miRNAs expressed by EC cells and in OSC samples exists despite their different phenotypes. EC is of germ cell origin whilst OSC is of epithelial origin. However, morphologically, EC is composed of primitive epithelial cells, which may explain the similarities reported here. It may also be related to tissue-specific expression or reflect a temporal relationship in terms of degree of dedifferentiation Regulation of miRNA biosynthesis and mature miRNA expression in these diverse samples indicates the importance of these mechanisms to ovarian malignancy generally. More than 80% of tumour-specific miRNAs were expressed in 2102Ep cells. This clearly indicates that miRNA regulation in 2102Ep cells is highly relevant to tumour samples, more relevant than miRNA regulation in tumour samples is to 2102Ep cells. Many of these miRNAs have reported associations with malignancy. Stem cells represent a small proportion of a well-differentiated tumour. In contrast, 2102Ep cells generate a malignant tumour *in vivo *that is almost completely EC cells [11,14], while melanoma contains a high proportion of stem cells [46]. Thus it is not surprising that highly aggressive 2102Ep cells are more relevant to tumour samples than NTera2 cells.

In this study we have identified two 2102Ep-specific mechanisms. A group of 21 miRNAs are constantly expressed, half of which are OSC-specific. The functional significance of this overlap is suggested by their validated targets. For example, miR-224 targets apoptosis inhibitor 5 (Api5) while miR-503 suppresses cyclinD1 [47,48]. 2102Ep cells respond to RA treatment via a second specific mechanism that is independent of NTera2 mechanisms and has not been previously demonstrated. Group 3 miRNAs are alternatively regulated in each differentiated cell type. This represents a 2102Ep mechanism that, in response to differentiation, acts in the exact opposite fashion to NTera2 cells. Approximately 62% of Group 3 miRNAs were OSC-specific, the largest overlap observed between EC cells and OSC samples. Group 3 miRNAs represent a key target group for future analysis. It is tempting to postulate that this mechanism may facilitate counteraction of differentiation to some extent, a possibility that will be assessed through ongoing analysis. miR-137 is an interesting example as it is expressed in only differentiated 2102Ep cells and in undifferentiated NTera2 cells and is associated with stemness and malignancy [49]. miR-137 is downregulated in OSC samples, indicating complex regulation. The identification of a fourth group of miRNAs is potentially highly relevant to our understanding of tumourigenesis from 2102Ep cells. Group 4 miRNAs are altered upon RA treatment of 2102Ep cells. In contrast, Group 4 miRNAs are not altered in NTera2 cells. This indicates that 2102Ep cells can regulate a specific miRNA response to this differentiation signal. Group 4 miRNAs displayed the lowest overlap with OSC samples. This suggests that Group 4 miRNAs are highly relevant to 2102Ep cells. It is possible that Group 4 miRNAs may act against differentiation to contribute to the high grade phenotype, a possibility that is being actively assessed.

## Conclusion

The highly malignant phenotype of 2102Ep EC cells employs a three pronged mechanism of miRNA regulation involving miRNA biosynthesis, levels of mature miRNA expression and alternative expression of miRNAs in response to differentiation. This miRNA regulation is associated with the ability of 2102Ep cells to avoid differentiation to generate high-grade tumours and that is relevant to tumour samples. These miRNAs are either similarly or alternatively expressed during tumourigenesis. As the precise mechanisms of miRNA targeting are still being elucidated, it is possible that miRNAs expressed in 2102Ep cells may play similar or diverse roles in OSCs. Due to their association with high-grade progenitor cells and tumours, Group 3 and 4 miRNAs are of particular relevance to future analysis.

## Abbreviations

Afp: Alpha fetal protein; CSC: Cancer stem cell; EC: Embryonal carcinoma; Eno3: Enolase 3; FFPE: Formalin-fixed paraffin-embedded; mRNA: Messenger RNA; miRNA: MicroRNA; Ncam1: Neural cell adhesion molecule 1; OSC: Ovarian serous adenocarcinoma; qPCR: Quantitative polymerase chain reaction; RA: Retinoic Acid RT: Reverse transcription; UPGM: Unweighted pair group method; UTR: Untranslated region.

## Competing interests

The authors declare that they have no competing interests.

## Authors' contributions

MFG, RJF and SAE designed the experiment, analyzed and interpreted data and were the primary authors of the manuscript. SAE and RJF conducted the experiment and data analysis. RJF (pathology), MFG, YMS, SFV (cancer stem cell biology) and MFG, MYL (stem cell biology) contributed to data interpretation. JKM designed figures and contributed to the preparation of the manuscript. PCS contributed to data analysis and experimental design. SOT and AL conducted experimental work. KD, JL and STA aided experimental design and procedure. KL contributed to technical interpretation of the data. CMM (tumour biology) and OS (pathology) critically analyzed the manuscript. JOL directly supervised the study. All authors read and approved the final manuscript.

## Supplementary Material

Additional file 1 **miRNAs in undifferentiated EC cells**. miRNAs expressed in each undifferentiated cell type and their chromosomal clustering are listed. Additionally, the relative expression values of miRNAs in undifferentiated 2102Ep cells compared to undifferentiated NTera2 cells are detailed. Click here for file

Additional file 2 **Top ten miRNAs in undifferentiated 2102Ep EC cells compared to undifferentiated NTera2 cells and their associations with malignancy**. The top ten miRNAs up- and down-expressed in undifferentiated 2102Ep cells compared to undifferentiated NTera2 cells, their previous associations with malignancy and these references are detailed. Click here for file

Additional file 3 **miRNAs in differentiated EC cells: Group 1-4**. miRNAs expressed in differentiated cells were divided into four groups based on their expression patterns. The miRNAs expressed in each group and their chromosomal clustering is detailed. Click here for file

Additional file 4 **The association of Group 3 and 4 miRNAs with malignancy**. Group 3 and 4 miRNAs, their previous associations with malignancy and these references are detailed. Click here for file

Additional file 5 **Comparison of miRNAs in EC cells and OSC patient samples**. miRNAs expressed in OSC samples, their rankings, chromosomal clustering and overlap with EC cells are described. Click here for file
