# Supplementary material for: Regulation of microRNA biosynthesis and expression in 2102Ep embryonal carcinoma stem cells is mirrored in ovarian serous adenocarcinoma patients
Source: J Ovarian Res. 2009 Dec 16;2:19. doi: 10.1186/1757-2215-2-19 (PMC2805659; doi:10.1186/1757-2215-2-19)
Supplement: Additional file 1 — miRNAs in undifferentiated EC cells. miRNAs expressed in each undifferentiated cell type and their chromosomal clustering are listed. Additionally, the relative expression values of miRNAs in undifferentiated 2102Ep cells compared to undifferentiated NTera2 cells are detailed. [file 1757-2215-2-19-S1.PDF]

Supplementary Table 1. miRNAs in undifferentiated EC cells.

203 miRNAs were commonly expressed in undifferentiated Ntera2 and 2102Ep cells and are ranked and listed in the left panels. An additional 21 miRNAs were specifically expressed in 2102Ep cells and are listed in the centre panel. 18 miRNAs were downregulated and 134 upregulated in undifferentiated 2102Ep cells compared to undifferentiated Ntera2 cells and are listed with their relative expression values in the right panel. Locations on chromosome 19 and 14 are highlighted in red and blue respectively.

Common miRNAs

Ntera Undiff	2102 Undiff
miR-222	miR-191
miR-19b	miR-302a
miR-19a	miR-221
miR-103	miR-222
miR-17-5p	miR-17-3p
miR-135b	miR-103
miR-25	miR-19b
miR-130a	miR-92
miR-30c	miR-320
miR-106b	miR-135b
miR-135a	miR-30c
miR-23a	miR-193b
miR-15b	miR-19a
miR-23b	miR-34a
miR-191	miR-23a
miR-18a	miR-106a
miR-30b	miR-200c
miR-27a	miR-130a
miR-186	miR-339
miR-296	miR-17-5p
miR-125a	miR-15b
miR-30d	miR-27a
miR-27b	miR-182
miR-107	miR-135a
miR-302b	miR-23b
miR-210	miR-30b
miR-24	miR-18a
miR-125b	miR-99b
miR-130b	miR-106b
miR-21	miR-25
miR-128a	miR-324-3p
miR-20a	miR-27b
miR-22	miR-210
miR-30a-5p	miR-30d
miR-302d	miR-197
miR-92	miR-148a
miR-205	miR-342
miR-132	miR-24
miR-221	miR-107
miR-30a-3p	miR-365
miR-133a	miR-22
miR-302a*	miR-21
miR-302a	miR-127
miR-181c	miR-186
miR-183	miR-130b

2102Ep-Specific miRNAs

2102Ep Undiff miRNA
miR-196b
miR-213
miR-448
miR-449
miR-375
miR-10b
miR-224
**miR-376a**
**miR-379**
miR-409-3p
**miR-493**
**miR-498**
miR-499
miR-503
**miR-516-3p**
miR-517*
**miR-525**
**miR-525***
**miR-526a**
**miR-527**
miR-508

Ntera vs 2102Ep (Undiff)

miRNA Downreg	2102Ep Fold Change
miR-296	-53.2
miR-211	-18.1
miR-139	-7.9
miR-181b	-4.8
miR-199a	-4.1
miR-363	-3.5
miR-302C*	-2.9
miR-302b	-2.7
miR-19a	-2.6
miR-190	-2.5
miR-17-5p	-2.3
miR-25	-2.3
miR-100	-2.3
let-7e	-2.3
miR-214	-2.2
let-7i	-2.2
miR-212	-2.1
miR-29a	-2.1
**Upreg**	
miR-21	2.1
miR-126	2
miR-184	2.1
miR-30c	2.1
**miR-27a**	2.1
miR-219	2.1
miR-103	2.2
miR-422a	2.6
miR-152	2.3
miR-491	2.4
miR-34b	2.4
miR-200b	2.6
miR-30e-5p	2.9
miR-30e-3p	2.9
miR-22	2.9
**miR-376b**	3.2
miR-148b	3.4
miR-26a	3.7
miR-501	3.8
miR-500	4
**miR-154**	4.2
miR-26b	4.5
miR-502	4.5
miR-143	5.5
miR-199b	5.7

miR-320	miR-124a	miR-146a	5.8
miR-26a	miR-205	miR-205	6.2
let-7e	miR-18b	miR-16	6.3
miR-148b	miR-125b	miR-145	7.4
miR-194	miR-183	**miR-136**	8.2
miR-181b	miR-373	miR-362	9.1
miR-423	miR-125a	miR-218	9.4
miR-28	miR-149	**miR-485-3p**	9.8
miR-29c	miR-154*	**miR-369-5p**	10.1
miR-29a	miR-151	**miR-432**	10.7
miR-200c	miR-328	miR-31	11
miR-185	miR-372	miR-193a	11.1
miR-218	miR-15a	miR-183	11.7
miR-20b	miR-20a	**miR-299-5p**	13.7
miR-212	miR-324-5p	**miR-410**	15
miR-106a	miR-302b	miR-424	15.3
miR-152	miR-128a	miR-497	16.3
let-7a	miR-331	miR-452*	20.8
miR-16	miR-187	**miR-329**	21.3
miR-100	miR-30a-5p	miR-101	23.2
miR-214	miR-335	**miR-409-5p**	26.8
miR-139	miR-218	**miR-337**	29.4
miR-422b	miR-302d	**miR-494**	32.9
miR-145	miR-34c	miR-18a*	34.3
miR-184	miR-181d	**miR-368**	35.5
miR-367	miR-30a-3p	miR-509	36
miR-197	miR-26a	miR-96	40.1
miR-93	miR-9	miR-92	54.9
miR-126	miR-192	miR-129	67.6
let-7i	miR-299-5p	miR-191	70.6
miR-193b	miR-512-3p	miR-331	73.5
miR-146a	miR-16	**miR-487**	78.8
miR-339	miR-145	**miR-520d**	83.4
miR-99b	miR-148b	miR-328	87.3
miR-299-5p	miR-129	**miR-518a-2***	89.2
miR-154	miR-18a*	miR-382	95
miR-193a	miR-191*	miR-UL112-1	99.6
miR-143	miR-512-5p	**miR-519b**	105.9
miR-200b	miR-146b	miR-197	106.4
let-7d	miR-193a	miR-451	109.8
miR-26b	miR-181c	miR-95	114.2
miR-30e-5p	miR-132	miR-187	117.6
miR-342	miR-134	**miR-517b**	126.5
miR-324-3p	miR-371	**miR-518b**	139.3
miR-328	miR-146a	miR-200c	145.1
miR-34a	miR-302a*	**miR-181d**	150.1
miR-124a	miR-133a	miR-320	155.5
miR-302b*	miR-101	miR-149	155.6
miR-331	miR-152	miR-512-5p	156.64
miR-150	miR-20b	miR-124a	161.9
miR-149	miR-133b	miR-15a	169.3
miR-17-3p	miR-143	miR-192	169.3
miR-18a*	miR-154	**miR-99b**	177.8
miR-363	miR-184	miR-512-3p	189.3
miR-30e-3p	miR-194	miR-516-5p	189.5
miR-505	miR-29c	miR-342	194.8
miR-187	miR-423	miR-106a	207.8
miR-15a	miR-185	**miR-517a**	246.7
miR-199a	miR-422b	**miR-519d**	250.4
miR-126*	miR-26b	miR-133b	258.6

let-7b	miR-126	**miR-515-3p**	272.5
miR-101	miR-204	miR-339	281.5
miR-151	miR-367	**miR-519e***	290.5
miR-129	miR-517a	miR-151	315.1
miR-31	miR-296	miR-324-3p	317
miR-18b	miR-32	miR-489	337.8
miR-181d	let-7a	miR-324-5p	348.7
miR-500	miR-200b	miR-34c	353.2
miR-324-5p	let-7e	miR-221	385
miR-182	miR-31	miR-UL22A-1	388
miR-192	miR-140	**miR-495**	405.2
miR-512-3p	miR-30e-5p	miR-338	415.7
miR-512-5p	miR-28	miR-1	427.9
miR-34c	miR-203	**miR-99a**	437
miR-127	miR-517b	let-7g	464.6
miR-148a	miR-29a	**miR-520c**	484.7
miR-365	miR-516-5p	miR-18b	486
miR-504	miR-99a	miR-340	492.5
miR-345	miR-212	miR-193b	500.8
miR-485-3p	let-7g	**miR-520f**	538.2
miR-455	miR-93	**miR-520e**	606.8
miR-501	miR-100	miR-146b	613.3
miR-422a	miR-30e-3p	miR-191*	635.1
miR-373	miR-214	**miR-519e**	667.1
miR-219	miR-340	miR-204	682.9
miR-362	miR-518b	miR-33	713
miR-34b	miR-181b	miR-140	838.2
miR-374	let-7i	miR-203	851.6
miR-133b	miR-338	**miR-521**	881.1
miR-191*	miR-519d	miR-34a	928.7
miR-146b	miR-495	miR-188	995.1
miR-491	miR-188	miR-302a	997.1
miR-9	miR-505	miR-9	1055.7
miR-517b	let-7d	miR-32	1324
miR-497	miR-489	miR-9*	1419.9
miR-517a	miR-520f	**miR-127**	1754.9
miR-211	miR-33	**miR-134**	1999.4
miR-424	miR-302b*	**miR-520a***	2006.4
miR-136	miR-500	miR-365	2130.7
miR-516-5p	miR-515-3p	**miR-373**	2378.6
miR-326	miR-126*	miR-148a	2399
miR-335	miR-485-3p	**miR-371**	2835
miR-372	miR-UL22A1	miR-182	3000.4
miR-518b	miR-520e	**miR-519c**	3571.2
miR-329	miR-520c	miR-335	4500.4
miR-452*	miR-9*	**miR-518c**	6371.7
miR-204	miR-150	miR-17-3p	6675.6
miR-134	miR-139	**miR-372**	8647.5
miR-154*	let-7b	**miR-154***	15922.9
miR-376b	miR-362		
miR-99a	miR-518c		
miR-371	miR-UL1121		
miR-519d	miR-497		
let-7g	miR-521		
miR-140	miR-1		
miR-203	miR-519e*		
miR-190	miR-501		
miR-UL112-1	miR-519b		
miR-32	miR-424		
miR-96	miR-329		

miR-340	miR-520a*
miR-338	miR-452*
miR-515-3p	miR-363
miR-489	miR-422a
miR-495	miR-519c
miR-494	miR-96
miR-519b	miR-199a
miR-409-5p	miR-519e
miR-520f	miR-136
miR-502	miR-345
miRUL22A-1	miR-219
miR-302C*	miR-382
miR-509	miR-34b
miR-33	miR-455
miR-432	miR-494
miR-520c	miR-491
miR-382	miR-520d
miR-188	miR-487
miR-519e*	miR-518a-2*
miR-200a*	miR-504
miR-520e	miR-409-5p
miR-487	miR-509
miR-520d	miR-374
miR-199b	miR-326
miR-410	miR-376b
miR-1	miR-368
miR-518a-2*	miR-432
miR-368	miR-410
miR-521	miR-502
miR-9*	miR-199b
miR-519e	miR-451
miR-520a*	miR-337
miR-337	miR-200a*
miR-518c	miR-190
miR-451	miR-95
miR-519c	miR-211
miR-369-5p	miR-302C*
miR-95	miR-369-5p
