# Supplementary material for: Regulation of microRNA biosynthesis and expression in 2102Ep embryonal carcinoma stem cells is mirrored in ovarian serous adenocarcinoma patients
Source: J Ovarian Res. 2009 Dec 16;2:19. doi: 10.1186/1757-2215-2-19 (PMC2805659; doi:10.1186/1757-2215-2-19)
Supplement: Additional file 2 — Top ten miRNAs in undifferentiated 2102Ep EC cells compared to undifferentiated NTera2 cells and their associations with malignancy. The top ten miRNAs up- and down-expressed in undifferentiated 2102Ep cells compared to undifferentiated NTera2 cells, their previous associations with malignancy and these references are detailed. [file 1757-2215-2-19-S2.PDF]

Supplementary Table 2. Top ten miRNAs in undifferentiated EC cells and their associations with malignancy.

The relative expression of top up- and downregulated miRNAs in undifferentiated 2102Ep cells compared to Ntera2 cells. Comparative analysis was carried out using the 2^{-ddCt} method (Livak and Schmittgen, 2001). Expression levels for miRNAs in undifferentiated Ntera2 cells were equated to 1 and levels in undifferentiated 2102Ep cells are presented proportionally to this.

**miRNA**	**Relative Expression**	**Malignancy**	**miRNA**	**Relative Expression**	**Malignancy**
**Upregulated**			**Downregulated**		
**miR-9**	1055.7	Lung [66]<br>Breast [49]	**miR-296**	-53.2	
**miR-32**	1324.0	Lung [66]<br>Prostate [56]	**miR-211**	-18.1	Oral [SR8]
**miR-9***	1419.0	Lung [66]<br>Breast [45]	**miR-139**	-7.4	Lung [66]<br>Tongue [65]
**miR-127**	1754.9	Breast [SR1]<br>Leukaemia [SR2]	**miR-181b**	-4.89	Neural [SR9]<br>Colorectal [SR10]
**miR-134**	1999.4	Tongue [66]	**miR-199a**	-4.1	Ovary [33]<br>Lung [66]
**miR-520a***	2006.4		**miR-363**	-3.5	Lymphoma [80]
**miR-365**	2130.7	Breast [SR1]	**miR-302C***	-2.9	
**miR-373**	2378.6	Leukaemia [51]<br>Prostate [SR3]	**miR-302b**	-2.7	Embryonal Carcinoma [SR11]
**miR-148a**	2399.0	Metastasis [SR4]<br>Leukaemia [SR5]	**miR-19a**	-2.6	Lymphoma [52]<br>Lung [53]
**miR-371**	2835.0		**miR-190**	-2.5	Pancreas [SR12]<br>Liver [SR13]
**miR-182**	3000.4	Prostate [65]<br>Melanoma [SR6]	**miR-17-5p**	-2.4	Leukaemia [55]<br>Colorectal [57]
**miR-519c**	3571.2		**miR-25**	-2.3	Gastric [60]

**miR-335**	4500.4	Ovary [58]<br>Myeloma [74]	**miR-100**	-2.3	Ovary [37]<br>Prostate [SR14]
**miR-518c**	6371.7	Tumorigenesis [63]	**let-7e**	-2.3	Ovary [61]<br>Tumorigenesis [63]
**miR-17-3p**	6675.6	Lymphoma [52]<br>Colorectal [62]	**miR-214**	-2.2	Ovary [38]
**miR-372**	8647.5	Germ Cell [SR7]<br>Tongue [91]	**let-7i**	-2.2	Ovary [39]
**miR-154***	15922.9	Leukaemia [SR2]<br>Tongue [66]	**miR-212**	-2.1	
			**miR-29a**	-2.1	Metastasis [SR15]<br>Leukaemia [SR15]

Supplementary Table 3 References

SR1. Yan LX, Huang XF, Shao Q, Huang MY, Deng L, Wu QL, Zeng YX, Shao JY: **MicroRNA miR-21 overexpression in human breast cancer is associated with advanced clinical stage, lymph node metastasis and patient poor prognosis.** *RNA* 2008, **14**(11):2348-60.

SR2. Dixon-McIver A, East P, Mein CA, Cazier JB, Molloy G, Chaplin T, Andrew Lister T, Young BD, Debernardi S: **Distinctive patterns of microRNA expression associated with karyotype in acute myeloid leukaemia**. *PLoS ONE* 2008, **14**(5):e2141.

SR3. Yang K, Handorean AM, Iczkowski KA: **MicroRNAs 373 and 520c Are Downregulated in Prostate Cancer, Suppress CD44 Translation and Enhance Invasion of Prostate Cancer Cells in vitro**. *Intl J Clinical Exper Path* 2009, **2**(4):361-9.

SR4. Lujambio A, Calin GA, Villanueva A, Ropero S, Sánchez-Céspedes M, Blanco D, Montuenga LM, Rossi S, Nicoloso MS, Faller WJ, Gallagher WM, Eccles SA, Croce CM, Esteller M: **A microRNA DNA methylation signature for human cancer metastasis**. *PNAS* 2008, **105**(36):13556-61.

SR5. Langer C, Radmacher MD, Ruppert AS, Whitman SP, Paschka P, Mrózek K, Baldus CD, Vukosavljevic T, Liu CG, Ross ME, Powell BL, de la Chapelle A, Kolitz JE, Larson RA, Marcucci G, Bloomfield CD: **High BAALC expression associates with other molecular prognostic markers, poor outcome, and a distinct gene-expression signature in cytogenetically normal patients younger than 60 years with acute myeloid leukemia: a Cancer and Leukemia Group B (CALGB) study.** *Blood* 2008, **111**(11):5371-9.

SR6. Segura MF, Hanniford D, Menendez S, Reavie L, Zou X, Alvarez-Diaz S, Zakrzewski J, Blochin E, Rose A, Bogunovic D, Polsky D, Wei J, Lee P, Belitskaya-Levy I, Bhardwaj N, Osman I and Hernando E. **Aberrant miR-182 expression promotes melanoma metastasis by repressing FOXO3 and microphthalmia-associated transcription factor.** *PNAS* 2009, **106**(6):1814-9.

SR7. Voorhoeve PM, le Sage C, Schrier M, Gillis AJ, Stoop H, Nagel R, Liu YP, van Duijse J, Drost J, Griekspoor A, Zlotorynski E, Yabuta N, De Vita G, Nojima H, Looijenga LH, Agami R: **A genetic screen implicates miRNA-372 and miRNA-373 as oncogenes in testicular germ cell tumors**. *Adv Exp Med Biol* 2007, **604**:17-46.

SR8. Chang KW, Liu CJ, Chu TH, Cheng HW, Hung PS, Hu WY, Lin SC: **Association between high miR-211 microRNA expression and the poor prognosis of oral carcinoma**. *J Dental Res*, **2008, 87(11):1063-8.**

SR9. Conti A, Aguennouz M, La Torre D, Tomasello C, Cardali S, Angileri FF, Maio F, Cama A, Germanò A, Vita G, Tomasello F: **miR-21 and 221 upregulation and miR-181b downregulation in human grade II-IV astrocytic tumors.** *J Neurooncol*, 2009, **93**(3):325-32.

SR10. Yantiss RK, Goodarzi M, Zhou XK, Rennert H, Pirog EC, Banner BF and Chen YT: **Clinical, pathologic, and molecular features of early-onset colorectal carcinoma**. *Am J Surg Pathol*, 2009, **33**(4):572-82.

SR11. Lee NS, Kim JS, Cho WJ, Lee MR, Steiner R, Gompers A, Ling D, Zhang J, Strom P, Behlke M, Moon SH, Salvaterra PM, Jove R, Kim KS: **miR-302b maintains "stemness" of human embryonal carcinoma cells by post-transcriptional regulation of Cyclin D2 expression.** *Biochem Biophys Res Comm*, 2008, **377**(2):434-40.

SR12. Zhang Y, Li M, Wang H, Fisher WE, Lin PH, Yao Q and Chen C: **Profiling of 95 microRNAs in pancreatic cancer cell lines and surgical specimens by real-time PCR analysis.** *World J Surg*, 2009, **33**(4):698-709.

SR13. Kutay H, Bai S, Datta J, Motiwala T, Pogribny I, Frankel W, Jacob ST and Ghoshal K: **Downregulation of miR-122 in the rodent and human hepatocellular carcinomas** *J Cell Biochem*, 2006, **99**(3):671-8.

SR14. Leite KR, Sousa-Canavez JM, Reis ST, Tomiyama AH, Camara-Lopes LH, Sañudo A, Antunes AA, Srougi M. **Change in expression of miR-let7c, miR-100, and miR-218 from high grade localized prostate cancer to metastasis**. *Urol Oncol*, 2009, **02**:2.

SR15. Zanette DL, Rivadavia F, Molfetta GA, Barbuzano FG, Proto-Siqueira R, Silva-Jr WA, Falcão RP, Zago MA: **miRNA expression profiles in chronic lymphocytic and acute lymphocytic leukemia**. *Braz J Med Biol Res*, 2007, **40**(11):1435-40.

SR16. Gebeshuber CA, Zatloukal K, Martinez J. **miR-29a suppresses tristetraprolin, which is a regulator of epithelial polarity and metastasis**. *EMBO Reports*, 2009, **10**(4):400-5.
