# Supplementary material for: Regulation of microRNA biosynthesis and expression in 2102Ep embryonal carcinoma stem cells is mirrored in ovarian serous adenocarcinoma patients
Source: J Ovarian Res. 2009 Dec 16;2:19. doi: 10.1186/1757-2215-2-19 (PMC2805659; doi:10.1186/1757-2215-2-19)
Supplement: Additional file 3 — miRNAs in differentiated EC cells: Group 1-4. miRNAs expressed in differentiated cells were divided into four groups based on their expression patterns. The miRNAs expressed in each group and their chromosomal clustering is detailed. [file 1757-2215-2-19-S3.PDF]

Supplementary Table 3. miRNAs in differentiated EC cells: Group 1-4.

miRNAs were arranged into four groups according to their expression patterns and are presented here, ranked according to their expression levels. Group 1 miRNAs are similarly altered in both differentiated cell types. Group 2 miRNAs are altered in differentiated Ntera2 cells but unaltered in differentiated 2102Ep cells. Group 3 miRNAs are altered in the opposite fashion in each differentiated cell type. miR-137 is downregulated in differentiated Ntera2 cells and upregulated in differentiated in differentiated 2102Ep cells. The remaining Group 3 miRNAs are upregulated in differentiated Ntera2 cells and downregulated in differentiating 2102Ep cells. Group 4 miRNAs are altered in differentiated 2102Ep cells but unaltered in Ntera2 cells. In each group miRNAs downregulated in OSC samples are shown in italics and those upregulated in bold. Clustering to chromosomes 19 and 14 is highlighted in red and blue respectively.

Group1 miRNA	Group2 miRNA	Group3 miRNA	Group4 miRNA
miR-507	miR-122a	*miR-137*	miR-518c*
miR-142-5p	miR-515-5p	miR-32	*miR-153*
miR-520b	miR-182*	**miR-320**	*let-7g*
**miR-522**	miR-199a*	**miR-324-3p**	miR-504
miR-299-3p	**miR-7**	miR-338	*miR-362*
miR-361	miR-206	*miR-34c*	*miR-17-3p*
miR-520g	miR-373*	miR-133b	miR-511
miR-490	*miR-380-3p*	miR-15a	miR-193b
miR-485-3p	miR-484	**miR-302a**	*miR-455*
miR-520e	**miR-301**	*miR-9**	miR-431
miR-516-5p	miR-519e*	*miR-203*	*miR-154**
miR-337	miR-520f	*miR-204*	*UL112-1*
miR-211	**miR-519d**		**miR-31**
**miR-184**	miR-519b		miR-302c*
miR-99a	miR-372		**miR-512-3p**
**miR-10a**	miR-302b		*miR-376b*
	miR-96		miR-155
	miR-409-5p		miR-25
	miR-302b*		*miR-199a*
	**miR-518a-2***		miR-326
	miR-519e		miR-518e
	**miR-93**		miR-487
	miR-519c		miR-19a
	miR-139		miR-199b
	miR-520d		miR-363
	miR-367		miR-129
	*miR-520a**		*miR-433*
	miR-371		miR-425
	**miR-183**		miR-105
	miR-329		
	miR-518b		
	**miR-373**		
	miR-517b		
	miR-190		
	miR-148a		
	miR-133a		
	let-7b		
	miR-28		
	miR-191		
	miR-92		

miR-UL22A-1
miR-214
**miR-187**
miR-342
**miR-200a***
miR-34a
miR-148b
**miR-218**
miR-494
miR-331
miR-146b
miR-30a-3p
miR-505
miR-192
miR-145
miR-221
miR-149
miR-22
miR-205
miR-328
miR-151
miR-451
miR-146a
miR-324-5p
miR-9
miR-339
*miR-18a**
miR-369-5p
miR-134
miR-382
miR-424
miR-340
miR-18b
miR-489
miR-1
miR-33
miR-140
miR-191*
miR-188
miR-99b
miR-509
miR-219
miR-335
let-7c
