# Supplementary material for: Regulation of microRNA biosynthesis and expression in 2102Ep embryonal carcinoma stem cells is mirrored in ovarian serous adenocarcinoma patients
Source: J Ovarian Res. 2009 Dec 16;2:19. doi: 10.1186/1757-2215-2-19 (PMC2805659; doi:10.1186/1757-2215-2-19)
Supplement: Additional file 4 — The association of Group 3 and 4 miRNAs with malignancy. Group 3 and 4 miRNAs, their previous associations with malignancy and these references are detailed. [file 1757-2215-2-19-S4.PDF]

Supplementary Table 4. The association of Group 3 and 4 miRNAs with malignancy.

**Group 3 miRNA**	**Malignancy**	**Group 4 miRNA**	**Malignancy**
	Glioblastoma [86]		Tumorigenesis [63]
miR-137	Melanoma [87]	miR-518c*	
	Lung [66]		
miR-32	Prostate [58]	miR-153	
	Tumorigenesis [63]		Lung [77]
miR-320		let-7g	Colon [78]
miR-324-3p		miR-504	
miR-338	Tongue [65]	miR-362	
	Leukaemia [SR17]		Lymphoma [52]
miR-34c	Lung [66]	miR-17-3p	Colorectal [62]
miR-365	Breast [SR1]	miR-511	Liver [92]
	Bladder [93]		Endometrioid [79]
miR-133b	Gastric [60]	miR-193b	
	Leukaemia [56]		
miR-15a	Lymphoma [52]	miR-455	
miR-302a		miR-431	
	Lung [66]		Leukaemia [SR2]
miR-9*	Breast [46]	miR-154*	Tongue [65]
	Colorectal [SR10]		
miR-203	Ovary [38]	UL112-1	
			Colorectal [57]
		miR-31	Gastric [60]
		miR-302c*	
		miR-512-3p	
		miR-376b	Breast [46]
		miR-155	
		miR-25	Gastric [60]
			Ovary [SR18]
		miR-199a	Lung [66]
		miR-326	
		miR-518e	
		miR-487	Liver [SR19]
			Lymphoma [52]
		miR-19a	Lung [53]
			Medullablastoma [79]
		miR-199b	Leukaemia [51]
		miR-363	Lymphoma [80]
		miR-129	Gastric [81]
		miR-433	
		miR-425	Glioblastoma [86]
		miR-105	Multiple [87]

Supplementary Table 4 References

SR17. Pigazzi M, Manara E, Baron E, Basso G. **miR-34b targets cyclic AMP-responsive element binding protein in acute myeloid leukemia.** *Cancer Res* 2009, **69**(6):2471-8.

SR18. Nam EJ, Yoon H, Kim SW, Kim H, Kim YT, Kim JH, Kim JW, Kim S. **MicroRNA expression profiles in serous ovarian carcinoma.** *Clin Cancer Research* 2008, **14**(9):2690-5.

SR19. Silva M, Moya A, Berenguer M, Sanjuan F, López-Andujar R, Pareja E, Torres-Quevedo R, Aguilera V, Montalva E, De Juan M, Mattos A, Prieto M, Mir J: **Expanded criteria for liver transplantation in patients with cirrhosis and hepatocellular carcinoma.** *Liver Transplant* 2008, **14**(10): 1449-60.
