# Supplementary material for: Regulation of microRNA biosynthesis and expression in 2102Ep embryonal carcinoma stem cells is mirrored in ovarian serous adenocarcinoma patients
Source: J Ovarian Res. 2009 Dec 16;2:19. doi: 10.1186/1757-2215-2-19 (PMC2805659; doi:10.1186/1757-2215-2-19)
Supplement: Additional file 5 — Comparison of miRNAs in EC cells and OSC patient samples. miRNAs expressed in OSC samples, their rankings, chromosomal clustering and overlap with EC cells are described. [file 1757-2215-2-19-S5.PDF]

Supplementary Table 5. Comparison of miRNAs in EC cells and OSC patient samples.

159 miRNAs (37 upregulated and 122 downregulated) were specifically expressed in Ovarian Serous Carcinoma (OSC) samples compared to normal ovary. These are ranked and are listed below. 11 of the 203 (55%) miRNAs commonly expressed by both undifferentiated EC cell types were OSC-specific. Of this 55%, 87 miRNAs were upregulated and 24 downregulated. Finally, of the 21 2102Ep-specific miRNAs identified in this study, seven were downregulated and three upregulated in OSC samples, which are listed below. Locations on chromosome 19 and 14 are highlighted in red and blue respectively.

miRNA UpReg	Undiff EC & Up in OSC	miRNA DownReg	Undiff EC & Down in OSC	2102Ep & OSC
miR-10a	miR-130b	let-7a	let-7a	**Down**
miR-130b	miR-135b	let-7b	let-7b	miR-376a
miR-135b	miR-182	let-7c	let-7d	miR-379
miR-141	miR-183	let-7d	let-7e	miR-493
miR-182	miR-184	let-7e	let-7g	miR-527
miR-183	miR-187	let-7f	let-7i	miR-508
miR-184	miR-18a	let-7g	miR-1	miR-503
miR-187	miR-200b	let-7i	miR-100	miR-10b
miR-18a	miR-200c	miR-1	miR-101	**Up**
miR-200a	miR-203	miR-100	miR-125a	miR-213
miR-200b	miR-20b	miR-101	miR-125b	miR-224
miR-200c	miR-218	miR-10b	miR-126*	miR-499
miR-203	miR-31	miR-125a	miR-127	
miR-20b	miR-338	miR-125b	miR-130a	
miR-213	miR-34b	miR-126*	miR-132	
miR-218	miR-34c	miR-127	miR-133a	
miR-223	miR-373	miR-130a	miR-133b	
miR-224	miR-422a	miR-132	miR-134	
miR-301	miR-489	miR-133a	miR-136	
miR-31	miR-512-3p	miR-133b	miR-140	
miR-338	miR-518a-2*	miR-134	miR-143	
miR-34b	miR-519d	miR-136	miR-145	
miR-34c	miR-93	miR-137	miR-148a	
miR-373	miR-95	miR-140	miR-151	
miR-422a	miR-130b	miR-143	miR-152	
miR-429		miR-145	miR-154*	
miR-483		miR-148a	miR-17-3p	
miR-499		miR-151	miR-186	
miR-512-3p		miR-152	miR-188	
miR-518a-2*		miR-153	miR-18a*	
miR-519d		miR-154*	miR-190	
miR-522		miR-17-3p	miR-192	
miR-7		miR-186	miR-193a	
miR-93		miR-188	miR-199a	
miR-95		miR-189	miR-204	
miR-10a		miR-18a*	miR-20a	

miR-130b	miR-190	miR-212
miR-135b	miR-192	miR-214
miR-141	miR-193a	miR-219
miR-182	miR-195	miR-222
miR-183	miR-199a	miR-23b
miR-184	miR-202	miR-26a
miR-187	miR-202*	miR-26b
miR-18a	miR-204	miR-27b
miR-200a	miR-20a	miR-28
miR-200b	miR-212	miR-299-5p
miR-200c	miR-214	miR-29a
miR-203	miR-216	miR-30b
miR-20b	miR-219	miR-30c
miR-213	miR-222	miR-30e-3p
miR-218	miR-23b	miR-30e-5p
miR-223	miR-26a	miR-32
miR-224	miR-26b	miR-328
miR-301	miR-27b	miR-329
miR-31	miR-28	miR-33
miR-338	miR-299-3p	miR-337
miR-34b	miR-133a	miR-299-5p
miR-34c	miR-29a	miR-342
miR-373	miR-29b	miR-345
miR-422a	miR-30b	miR-34a
miR-429	miR-30c	miR-362
miR-483	miR-30e-3p	miR-368
miR-499	miR-30e-5p	miR-369-5p
miR-512-3p	miR-32	miR-374
miR-518a-2*	miR-323	miR-376b
miR-519d	miR-328	miR-382
miR-522	miR-329	miR-410
miR-7	miR-33	miR-424
miR-93	miR-337	miR-432
miR-95	miR-339	miR-451
miR-10a	miR-342	miR-455
miR-130b	miR-345	miR-489
miR-135b	miR-34a	miR-494
miR-141	miR-361	miR-495
miR-182	miR-362	miR-497
miR-183	miR-368	miR-500
miR-184	miR-369-5p	miR-501
miR-187	miR-374	miR-502
miR-18a	miR-376a	miR-505
miR-200a	miR-376b	miR-509
miR-200b	miR-377	miR-517b
miR-200c	miR-379	miR-520a*
miR-203	miR-380-3p	miR-520c
miR-20b	miR-380-5p	miR-9*

miR-213
miR-218
miR-223
miR-224
miR-301
miR-31
miR-338
miR-34b
miR-34c
miR-373
miR-422a
miR-429
miR-483
miR-499
miR-512-3p
miR-518a-2*
miR-519d
miR-522
miR-7
miR-93
miR-95
miR-10a
miR-130b
miR-135b
miR-141
miR-182
miR-183
miR-184
miR-187
miR-18a
miR-200a
miR-200b
miR-200c
miR-203
miR-20b
miR-213
miR-218
miR-223
miR-224
miR-301
miR-31
miR-338
miR-34b
miR-34c
miR-373
miR-422a
miR-429
miR-483

miR-381
miR-382
miR-383
miR-410
miR-424
miR-432
miR-433
miR-450
miR-451
miR-455
miR-485-5p
miR-486
miR-488
miR-489
miR-493
miR-494
miR-495
miR-497
miR-500
miR-501
miR-502
miR-503
miR-505
miR-506
miR-507
miR-508
miR-509
miR-517b
miR-520a*
miR-520c
miR-527
miR-9*
miR-92
miR-99a
miR-UL112-1
let-7a
let-7b
let-7c
let-7d
let-7e
let-7f
let-7g
let-7i
miR-1
miR-100
miR-101
miR-10b
miR-125a

miR-92
miR-99a
miR-UL112-1
let-7a
let-7b
let-7d
let-7e
let-7g
let-7i
miR-1
miR-100
miR-101
miR-125a
miR-125b
miR-126*
miR-127
miR-130a
miR-132
miR-133a
miR-133b
miR-134
miR-136
miR-140
miR-143

miR-499
miR-512-3p
miR-518a-2*
miR-519d
miR-522
miR-7
miR-93
miR-95
miR-10a
miR-130b
miR-135b
miR-141
miR-182
miR-183
miR-184
miR-187
miR-18a
miR-200a
miR-200b
miR-200c
miR-203
miR-20b
miR-213
miR-218
miR-223
miR-224
miR-301

miR-125b
miR-126*
miR-127
miR-130a
miR-132
miR-133a
miR-133b
miR-134
miR-136
miR-137
miR-140
miR-143
miR-145
miR-148a
miR-151
miR-152
miR-153
miR-154*
miR-17-3p
miR-186
miR-188
miR-189
miR-18a*
miR-190
miR-192
miR-193a
